# The impact of episodic screening interruption: COVID-19 and population-based cancer screening in Canada

**DOI:** 10.1177/0969141320974711

**Published:** 2020-11-26

**Authors:** Jean HE Yong, James G Mainprize, Martin J Yaffe, Yibing Ruan, Abbey E Poirier, Andrew Coldman, Claude Nadeau, Nicolas Iragorri, Robert J Hilsden, Darren R Brenner

**Affiliations:** 1 Independent Consultant, Toronto, Canada; 2 282299 Sunnybrook Research Institute, Toronto, Canada; 3 Departments of Medical Biophysics and Medical Imaging, University of Toronto, Toronto, Canada; 4 Department of Cancer Epidemiology and Prevention Research, 3146 Alberta Health Services, Calgary, Canada; 5 British Columbia Cancer Research, Vancouver, Canada; 6 6360 Statistics Canada, Ottawa, Canada; 7 University of Toronto, Toronto, Canada; 8 Forzani & MacPhail Colon Cancer Screening Centre, 3146 Alberta Health Services, Calgary, Canada; 9 Departments of Medicine and Community Health Sciences, Cumming School of Medicine, 2129 University of Calgary, Calgary, Canada; 10 Departments of Oncology and Community Health Sciences, Cumming School of Medicine, 2129 University of Calgary, Calgary, Canada

**Keywords:** COVID-19, mammography screening, colo-rectal cancer screening, interruption of screening

## Abstract

**Background:**

Population-based cancer screening can reduce cancer burden but was interrupted temporarily due to the COVID-19 pandemic. We estimated the long-term clinical impact of breast and colorectal cancer screening interruptions in Canada using a validated mathematical model.

**Methods:**

We used the OncoSim breast and colorectal cancers microsimulation models to explore scenarios of primary screening stops for 3, 6, and 12 months followed by 6–24-month transition periods of reduced screening volumes. For breast cancer, we estimated changes in cancer incidence over time, additional advanced-stage cases diagnosed, and excess cancer deaths in 2020–2029. For colorectal cancer, we estimated changes in cancer incidence over time, undiagnosed advanced adenomas and colorectal cancers in 2020, and lifetime excess cancer incidence and deaths.

**Results:**

Our simulations projected a surge of cancer cases when screening resumes. For breast cancer screening, a three-month interruption could increase cases diagnosed at advanced stages (310 more) and cancer deaths (110 more) in 2020–2029. A six-month interruption could lead to 670 extra advanced cancers and 250 additional cancer deaths. For colorectal cancers, a six-month suspension of primary screening could increase cancer incidence by 2200 cases with 960 more cancer deaths over the lifetime. Longer interruptions, and reduced volumes when screening resumes, would further increase excess cancer deaths.

**Conclusions:**

Interruptions in cancer screening will lead to additional cancer deaths, additional advanced cancers diagnosed, and a surge in demand for downstream resources when screening resumes. An effective strategy is needed to minimize potential harm to people who missed their screening.

## Introduction

The coronavirus disease 2019 (COVID-19) global pandemic has affected 215 countries with over 37 million cases and over one million deaths reported as of 10 October 2020.^1^ In Canada, there have been over 178,000 cases of COVID-19 and over 9500 deaths caused by COVID-19 between 25 January 2020 and 10 October 2020.^2^ As part of the pandemic response, all elective and non-urgent scheduled surgeries and medical procedures were cancelled across Canada. All population-based cancer screening programs were suspended. These measures were initiated to make acute care resources available for COVID-19 patients and to minimize person-to-person virus transmissions in clinical settings.

In Canada, colorectal cancer is expected to be the third most commonly diagnosed cancer in 2020 and the second leading cause of cancer-related death.^3^ Breast cancer is the most commonly diagnosed cancer and second highest cause of cancer death in women in Canada.^3^ Early detection of adenomas/colorectal cancers and breast cancers has been demonstrated to save lives and is cost-effective.^4^ Organized screening programs for breast cancer began in 1988 and by 2008 were operating in all parts of Canada except the northern territory of Nunavut. Organized colorectal screening programs began in Canada in 2007 and are now in place in most parts of the country.^5,6^ Although suspending cancer screening was deemed necessary in the initial phase of the pandemic response, the impact on patient outcomes is unclear.

As healthcare administrators begin the restoration of medical services, new challenges have emerged with initial uncertainty about the availability of personal protective equipment, concern regarding potential new waves of COVID-19 outbreak, and potentially lower volumes of medical procedures as a result of more stringent infection control and physical distancing measures.

We have estimated the potential impact of suspending colorectal and breast cancer primary screening for up to 12 months on cancer outcomes in the population of Canada using a mathematical simulation model. We also evaluated the impact of potential reduced procedural volumes in the return to screening services.

## Methods

We simulated the detection and progression of breast and colorectal cancer outcomes using the colorectal and breast cancer models in OncoSim (version 3.3.6), a cancer microsimulation modeling tool developed by The Canadian Partnership Against Cancer with support from Statistics Canada.^7^ OncoSim creates “histories” of one individual at a time to mimic the demographics of the Canadian population. The development, application, and validation of the OncoSim models have been described previously.^8–11^ The breast and colorectal cancer models projected similar cancer incidence and mortality trends to those in the Canadian Cancer Registry and reproduced the observed effects of cancer screening in randomized trials.^8,11^

### OncoSim-Breast cancer microsimulation model

OncoSim-Breast simulates the onset, growth, and spread of invasive tumors and ductal carcinoma in situ (DCIS) (Supplementary Figure 1). The simulation of natural history was calibrated to match Canadian cancer incidence and mortality data. The model has been described in detail earlier^11^ and used to study the impact of breast cancer interventions on all-cause mortality.^9^ Additional details on the model and validation are provided in Supplementary Tables 1 to 4 and Supplementary Figures 2 to 5. The natural history component was inspired by the University of Wisconsin breast cancer microsimulation model.^12,13^ OncoSim-Breast captures the benefits of screening on breast cancer survival using the lead time that was calibrated from observed survival data. Stage-specific outcomes were used to determine breast cancer-specific mortality. To account for geographic variation, the survival outcomes were adjusted for each province and territory using relative risks, calibrated to match province-specific cancer mortality data in the Canadian Cancer Registry. The effects of population increase and aging demographics are modeled, contributing to an annual increase in the number of breast cancers.

### OncoSim-Colorectal microsimulation model

OncoSim-Colorectal simulates the natural history and progression of adenomas and colorectal cancer. The OncoSim-Colorectal cancer model has been described in detail in an earlier publication.^14^ The model assumes that most colorectal cancers develop from adenomas; adenomas can progress in size from small (≤5 mm) to medium (6–9 mm) to large (≥10 mm), transform into stage I preclinical cancer, or regress (Supplementary Figure 6). The model assumes that screening can detect adenomas and colorectal cancers at a preclinical (no symptoms) stage. Through calibration, the model replicates the prevalence of adenomas in the literature and colorectal cancer incidence and mortality for the Canadian population. Stage-specific survival rates were calibrated to match the numbers expected from the Canadian Mortality Database. The model assumes that screen-detected cancers have better stage-specific cancer survival rates than those detected clinically. Additional model details are provided in Supplementary Tables 5 to 9 and Supplementary Figure 7.

### Scenarios

The simulation was restricted to an appropriately weighted combination of birth cohorts within the range 1920–2015 to reflect the age distribution of the Canadian public. Screening before the year 2020 was modeled to take place as per empirical dissemination patterns in each calendar year.

We simulated scenarios of no screening interruption (“no interruption”) and screening interruptions of 3, 6, or 12 months. When screening resumes, various transition periods (0–24 months) of operation at 50% reduced screening volumes were modeled (Table 1). Post-interruption, we assumed that screening volumes would either return to 100% of pre-COVID levels immediately or that screening would occur at 50% reduced volume for a transition period (six months to two years).

**Table 1.** Breast and colorectal cancer screening interruption scenarios. Numbers in cells represent the percentage of full screening volume occurring during the time intervals, indicated in the columns. Cells filled with dark gray denote the primary period of interruption.

	Time (months)						
	0–6		6–12	12–18	18–24	24–30	30–36
Scenario							
3-month interruption	0	100	100	100	100	100	100
6-month interruption	0		100	100	100	100	100
12-month interruption	0		0	100	100	100	100
6-month interruption + 6-month transition	0		50	100	100	100	100
6-month interruption + 12-month transition	0		50	50	100	100	100
6-month interruption + 24-month transition	0		50	50	50	50	100
12-month interruption + 12-month transition	0		0	50	50	100	100
12-month interruption + 24-month transition	0		0	50	50	50	50

## Breast cancer screening

We assumed that women at average risk receive biennial screening between the ages 50 and 74 and that women identified as higher risk (e.g. familial cancer, genetic carriers) receive annual screening between the ages 40 and 74 (see Supplementary Table 10). Recruitment and rescreening (retention) rates were set such that the participation rate in 2019 was 63.2% (at least one mammogram in 36 months) for women aged 50–74. For comparison, the participation rate in the Ontario Breast Screening program was 65.7% in 2011.^15^ We simulated a cohort of individuals born in 1920–2015 and tracked their lifetime outcomes; these individuals would be at screen-eligible age in 1994–2055. For example, in 2020, the model estimated 2.4 million women would be undergoing breast cancer screening, assuming no interruptions. The input data for screening (including age and lesion size effects on sensitivity) were calibrated to match observed data in the Canadian Breast Cancer Screening Database. The model also incorporates a probability of clinical detection (e.g. due to palpable lump, nipple discharge, skin alterations) of cancer for all women, screened and unscreened, that is tumor size dependent.

In the breast cancer screening interruption scenarios, we assumed screening resumes with the order of examinations the same as would have occurred in the absence of COVID-19, except with delay by the duration of the interruption and a possible recovery period with reduced screening volumes. For example, in the scenario where screening was interrupted for six months, those who missed screening in March 2020 would receive screening in September 2020.

## Colorectal cancer screening

Our colorectal cancer screening scenarios assumed that high-risk populations (first-degree relatives with colorectal cancer) aged 40–74 receive screening colonoscopy every five years, and average risk populations aged 50–74 receive a Fecal Immunochemical Test (FIT) screening every two years (see Supplementary Table 10). Persons with a positive FIT result are followed up with diagnostic colonoscopy. The model assumed overall participation rate of ∼43%, based on data from the Canadian Community Health Surveys. In the simulation cohort of individuals born in 1920–2015, the model estimated 2.25 million FIT and 143,700 colonoscopies would be performed in 2020, without screening interruptions. In the screening interruption scenarios, we assumed that both FIT and colonoscopy screening are interrupted; however, symptomatic individuals still receive diagnostic examinations and surveillance colonoscopies. To simplify the modeling, we assumed previous screening participants who missed screening during interruptions or recovery would skip to the next screening interval. For example, if a person was due for a recall for biennial FIT screening in March 2020 and screening was not available to the person at that time, the test would occur in March 2022. To estimate the potential clinical benefits of a best-case scenario for reducing missed screens, we also modeled a (“catch-up”) scenario where individuals who missed screening during a six-month interruption would receive screening immediately when programs resume, with no delay in screening for those who are due for screening after programs have resumed.

### Outcomes

For breast cancer, we estimated the incidence and excess breast cancer incidence over time, the lifetime additional advanced stage (stages III and IV) breast cancers, deaths and life-years lost, if screening programs are interrupted for up to 12 months. For colorectal cancer, we estimated the number of undiagnosed advanced adenomas (polyp ≥10 mm) and colorectal cancers for the period that primary screening was suspended, the incidence of colorectal cancer over time, and the excess lifetime colorectal cancer incidence, mortality, and life-years lost, if screening programs were interrupted for up to 12 months. Confidence intervals (95%) were estimated from measurements of mean and variance of parameters extracted from 12 subsample outputs for each simulation run with the assumption that values are normally distributed.

## Results

### Impact of breast cancer screening interruption

**Figure 1.**
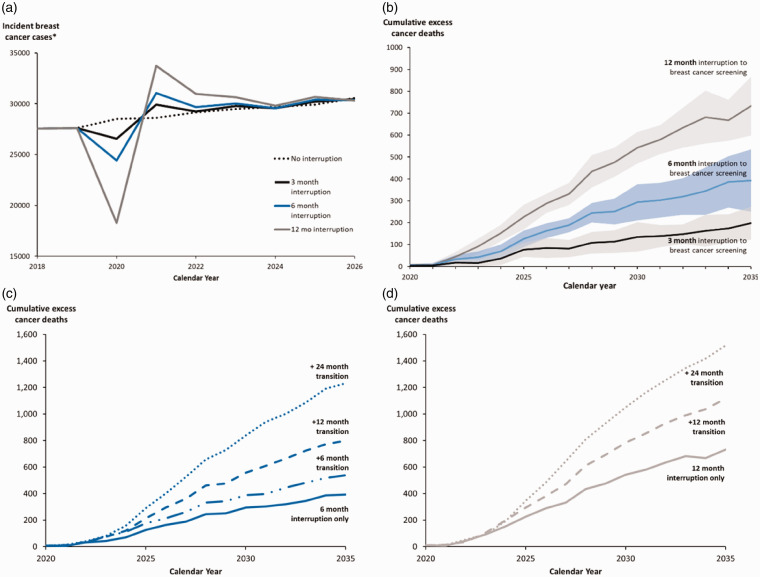
(a) Projected number of incident cases in Canada (*invasive cancer and DCIS combined) in 2018–2026 for the no interruption scenario, 3-, 6-, and 12-month interruption scenarios. (b) Projected cumulative excess breast cancer deaths (95% confidence intervals) for 3-, 6-, and 12-month interruptions. (c) Projected cumulative excess breast cancer deaths for 6-month interruption followed by 6-, 12-, and 24-months of 50% reduced capacity transition periods. (d) Projected cumulative excess breast cancer deaths for 12-month interruption followed by 12- and 24-months of 50% reduced capacity transition periods.

Our simulation models suggest that a three-month interruption of breast cancer screening due to COVID-19 would result in 644,000 fewer screens performed in Canada in 2020. In turn, the diagnosed incident breast cancer cases (invasive and DCIS) would drop from 28,500 to 26,600, a 7% decrease. A six-month interruption would result in a drop to 24,400 diagnosed cases, a 14% decrease. This is accompanied by an increase in non-screen-detected cancers of 550 and 1020 (10% and 19% increase) that year for three- and six-month interruptions, respectively, with the increase persisting for at least a year after screening resumes. After screening resumes, there would be a surge in screen-detected cases in 2021–2025 (Figure 1(a)).

A three-month service interruption would lead to 310 (95% confidence interval (CI): 220–390) more cancer cases detected at a late stage (Stage IIIA or higher) (Figure 3) and 110 more cancers deaths in 2020–2029 (Figure 1(b) to (d)). A six-month service interruption would lead to 670 of these later-stage cancer cases and 250 more cancer deaths (an increase of 0.48%) compared to no interruption (Table 2). The cumulative excess deaths following an interruption would continue to rise well beyond 2030 (Figure 1(b)). A six-month interruption would potentially result in 8000 (95% CI: 3500–12,000) life-years lost over the lifetime, assuming no catch-up strategy for those who missed their screening in 2020. Longer interruptions (12 months) would have a larger impact on the spike in diagnosed cancer cases (Figure 1(a)), stage shift, and cumulative excess cancer deaths (Figure 1(b)). After a 12-month interruption of breast cancer screening, 62% of the excess cancers at advanced stages would be diagnosed in the 60–74 age group.

**Table 2.** Estimated cumulative excess breast and colorectal cancer deaths in Canada for 6- and 12-month interruptions followed by operation at reduced (50%) volume for 0, 6, 12, or 24 months.

Cancer site	Year	Transition period (months)Excess cumulative deaths (% of baseline model)				Cumulative deaths (no interruption)
		0	6	12	24	
Breast		6-month interruption				
	2024	70 (0.27%)	110 (0.43%)	120 (0.46%)	160 (0.62%)	25,810
	2029	250 (0.48%)	340 (0.65%)	480 (0.92%)	730 (1.4%)	52,060
	2034	390 (0.49%)	520 (0.66%)	770 (0.97%)	1190 (1.5%)	79,280
		12-month interruption				
	2024	150 (0.58%)	–	200 (0.77%)	190 (0.74%)	25,810
	2029	480 (0.92%)	–	690 (1.3%)	930 (1.8%)	52,060
	2034	670 (0.85%)	–	1040 (1.3%)	1420 (1.8%)	79,280
Colorectal		6-month interruption				
	2024	130 (0.29%)	190 (0.43%)	220 (0.49%)	240 (0.54%)	44,630
	2029	450 (0.49%)	700 (0.76%)	930 (1.0%)	1150 (1.2%)	92,270
	2034	690 (0.48%)	1050 (0.73%)	1430 (0.99%)	1800 (1.2%)	144,210
		12-month interruption				
	2024	260 (0.58%)	–	300 (0.67%)	260 (0.58%)	44,630
	2029	930 (1.0%)	–	1350 (1.5%)	1800 (2.0%)	92,270
	2034	1360 (0.94%)	–	2090 (1.4%)	2880 (2.0%)	144,210

Our analyses suggest that persistent restrictions in screening volume post-interruption would lead to further excess cancer deaths (Figures 1(c) and (d) and Table 2). For example, in 2020–2029, the number of breast cancer deaths for a six-month interruption would increase from 250 expected breast cancer deaths with immediate restoration of screening (0 months transition) to 730 deaths (i.e. 1.4% increase above no interruption) for a 24-month transition at reduced screening volumes. Compared to the model prediction of 18,000 lives saved in 2020–2029 without screening interruption, this is a reduction in lives saved of 1.4% (0-month transition) and 4.0% (24-month transition).

### Impact of colorectal cancer screening interruption

**Figure 2.**
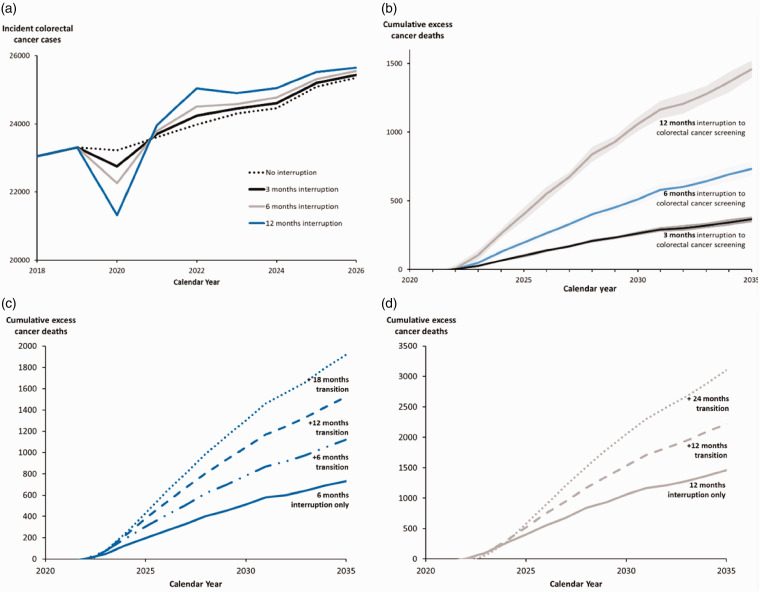
(a) Projected number of incident colorectal cancer cases in Canada in 2018–2026 for the no interruption scenario, 3-, 6-, and 12-month interruption scenarios. (b) Projected cumulative excess colorectal cancer deaths (95% confidence intervals) for 3-, 6-, and 12-month interruptions. (c) Projected cumulative excess colorectal cancer deaths for 6-month interruption followed by 6-, 12-, and 18-months of 50% reduced capacity transition periods. (d) Projected cumulative excess colorectal cancer deaths for 12-month interruption followed by 12- and 24-months of 50% reduced capacity transition periods.

**Figure 3.**
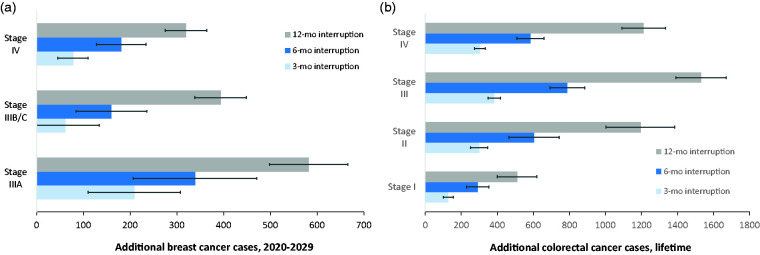
(a) Impact of interruption to mammography on additional breast cancers diagnosed between 2020 and 2029 at a later stage resulting from a 3-, 6-, and 12-month service interruption in Canada. (b) Lifetime impact of colorectal cancer screening interruptions on additional colorectal cancers, stratified by cancer stages, resulting from a 3-, 6-, and 12-month interruptions.

Without service interruption, we estimated that 68,000 colonoscopies would have been performed in the six months since March 2020 in Canada. The colorectal cancer model estimated that colorectal cancer screening without interruptions would avert 34,000 colorectal cancer deaths in 2020–2029. Similar to mammography, after screening resumes, there would be a surge in diagnosed cases in 2021–2025 (Figure 2(a)) and an increase in excess cancer deaths (Figure 2(b)). If screening were interrupted for six months, the opportunity for an earlier diagnosis of 19,000 adenomas and colorectal cancers would be missed; of these, about 9700 would be advanced adenomas and cancers. Over the lifetime, a three-month or six-month screening interruptions would lead to 1100 or 2200 more colorectal cancer cases respectively, with over 60% of the cases at an advanced stage (III or IV) (Figure 3(b)), and 480 or 960 more cancer deaths. This amounts to 31,100 (95% CI: 28,300–33,800) life-years lost for a six-month interruption.

Similar to breast cancer screening, longer interruptions (12 months) and transition periods have a larger impact (Figures 2(c) and (d) and Table 2). Under a 12-month interruption of colorectal cancer screening, 82% of the excess cancers at advanced stages would be diagnosed in the 60–74 age group. In 2020–2029, the number of deaths for a six-month interruption would increase from 450 (0.49%) of excess cancer deaths with immediate restoration of screening (0 months transition) to 1150 (1.2%) deaths if a 24-month transition period at reduced screening volumes followed the interruption (Table 2). Compared to the 34,000 lives expected to be saved in 2020–2029 without interruptions, this is a reduction in lives saved of 1.3% (0-month transition) and 3.3% (24-month transition). We simulated a crude catch-up scenario where after a six-month interruption in colorectal cancer screening, a catch-up ensues with no delay in tests originally scheduled to take place post interruption, but with a surge in testing so that missed screens occur immediately following the interruption. Compared to no catch-up, the excess colorectal cancer cases are reduced from 2200 to 310 and excess deaths reduced from 960 to 120.

## Interpretation

A short-term interruption of breast and colorectal cancer screening in Canada would lead to a surge in diagnosed cancer cases when screening resumes. Without adequate strategies to accommodate individuals who missed their screening, a six-month interruption would lead to ∼40,000 life-years lost. These results reinforce the importance of resuming cancer screening service levels quickly, while respecting infection prevention requirements.

Our analysis also showed that screening interruptions would increase the number of late-stage cancer cases. Treatment of more advanced cancers generally involves more use of systemic therapies, which could be associated with increased morbidity and higher costs.^16^ In a study of over 8000 treatments in the U.S., Blumen et al. found that the average reimbursed cost of breast cancer treatment was “$71,909, $97,066, $159,442 and $182,655 for disease stage 0, I/II, III, and IV, respectively.”^16^

Finally, our results suggest that the mortality impact depends both on the duration of interruption of screening and on how quickly and thoroughly screen-eligible individuals return to the recommended screening regimen. For this reason, it would be key to restore confidence both in the safety of participation in screening and its effectiveness. There are two important components to this. First, procedures for the safe flow of screening participants through facilities, especially waiting and changing areas, use of personal protective equipment, and hygiene must be established and enforced. Then, a well-designed knowledge translation and outreach plan to share this information with the public must be implemented. In their observational study of the impact of COVID-19 on screening volumes in Taiwan, Peng et al. made similar recommendations in their guidance for the safe restoration of screening.^17^

Sharpless has reported the results of a similar analysis in the US.^18^ Researchers used the CISNET cancer simulation models to estimate the impact of an interruption of screening for breast and colon cancer in the US. They predicted approximately 5300 additional breast cancer deaths and 4500 additional deaths due to colorectal cancer. When scaled by the difference of population between the US and Canada, the findings are remarkably similar to ours.

In our study, we investigated the effect of delays in screening, but assumed (as appears to have largely been the case) that subsequent procedures to obtain a definitive diagnosis would be carried out without delay. In a microsimulation study conducted in the United States, investigators focused on estimating changes in lifetime screening benefits when the intervals between screening and diagnosis for breast, colorectal, and cervical cancers were increased.^19^ Analyses were based on four timeframes for diagnostic testing after an abnormal screening result including immediate follow-up and at 3, 6, and 12 months. A six-month increase in diagnostic interval would reduce screening effectiveness resulting in fewer life-years gained per 1000 screened, fewer colorectal cancers prevented, and a distribution shift to higher stages at diagnosis for breast and colorectal cancers.^19^

The results of a crude colorectal cancer screening catch-up scenario showed that a large surge in screening volume when screening resumes could reduce the impact of screening interruptions on the excess colorectal cancer cases and cancer deaths. However, to accommodate the catch-up, programs would need to have excess colonoscopy capacity to accommodate greatly increased screening throughput. Moreover, to maintain the rescreen schedule, this surge would recur every two years (Supplementary Figure 8), although these peaks would broaden naturally as people schedule their subsequent screens.

As the daily number of COVID-19 incident cases decrease in each Canadian province, some jurisdictions have restored screening services while others will likely resume in the coming months. A surge in procedural volumes to catch-up with missed screens is generally not feasible for three reasons. First, most facilities were operating at full capacity prior to COVID and screening rates are limited by the availability of trained personnel (radiologists and technologists) and these individuals are in short supply in Canada. An increase in screening volumes would require some time (and funds) for hiring and training. Second, there is likely to be an increase in the volume of diagnostic procedures. These are given priority over screening and are carried out on the same imaging equipment by the same personnel. Finally, new safety requirements associated with physical spacing, sanitization, and personal protective equipment will add time to the workflow, affect scheduling, and limit the number of screening examinations that can be carried out per hour.

With long wait times for follow-up diagnostic procedures such as colonoscopy, already a barrier for patients before the COVID-19 pandemic, adequate prioritization strategies will become even more important in the coming months and years to ensure urgent and priority cases are triaged appropriately. For example, upon the restart of colorectal cancer screening, several provinces have recommended prioritizing the use of colonoscopies for those with symptoms or a positive FIT test. Additional financial investment to increase diagnostic capacity could also help alleviate wait times and health burden. However, given the financial toll imposed by COVID-19, additional funding is likely to be a challenge. Therefore, further modeling studies and innovative research-guided approaches will be essential to compare the risks and benefits of screening strategies to cope with reduced capacity in follow-up diagnostics.

### Limitations

This study has several limitations. First, although the models reproduce recent cancer trends in the Canadian Cancer Registry and screening effects in randomized trials, there are no data to validate the prediction of screening interruptions on long-term outcomes. Second, the validity of results depends on assumptions around the length of the screening delays, availability of health services when screening resumes, screening participant behaviors in trading off risks and benefits of screening during a pandemic after screening services are restored and cancer risk profiles of screening participants. We attempted to demonstrate the effect of the transition period on the long-term outcomes. Third, the impact of interruptions on long-term cancer outcomes depends on how individuals who missed their screening are called back for additional screening. Screening interruptions were simulated differently in the breast and colorectal cancer models because the models were built with different assumptions. Due to constraints of the colorectal cancer model, participants who missed colorectal cancer screening during the interruption and recovery periods would skip to the next screening interval; the impact of the interruption would be smaller if those individuals were able to receive colorectal cancer screening immediately after screening programs resumed. In contrast, when simulating breast cancer screening interruptions, those who missed screening were called back for screening when the program resumed. This led to a larger predicted impact on long-term colorectal cancer outcomes than in breast cancer. Fourth, in estimating the impact of interruption on breast cancer stage shift, we were not able to count the number of cases that were detected at a later stage due to interruptions. For example, the net number of additional Stage III cases is a combination of Stage II cases that progress to Stage III minus some Stage III cases that progress to stage IV. Fifth, we have likely underestimated the impact of screening interruptions on colon cancer deaths because we assumed surveillance and symptomatic colonoscopies were not affected in 2020–2021. Finally, we assumed that all abnormal findings receive adequate work-up procedures and subsequent treatment without delay related to the pandemic or its aftermath. Given the expected impact on health services, the assumption of no delay in diagnostic procedures is unlikely.

## Conclusion

A brief interruption in cancer screening will result in additional deaths due to breast and colon cancer and a shift toward diagnosis of more advanced cancers. It will also have an impact on downstream resources when screening resumes. Until screening volumes return to pre-COVID levels, interruptions could have a large adverse impact on long-term cancer outcomes. Further delays to re-starting screening or reduced volumes due to COVID-related regulations will have an additional impact. These data support the recommendations for immediate restoration of cancer screening when it is safe with adequate prioritization strategies to mitigate harms.

## Supplemental Material

sj-pdf-1-msc-10.1177_0969141320974711 - Supplemental material for The impact of episodic screening interruption: COVID-19 and population-based cancer screening in Canada Click here for additional data file. Supplemental material, sj-pdf-1-msc-10.1177_0969141320974711 for The impact of episodic screening interruption: COVID-19 and population-based cancer screening in Canada by Jean HE Yong, James G Mainprize, Martin J Yaffe, Yibing Ruan, Abbey E Poirier, Andrew Coldman, Claude Nadeau, Nicolas Iragorri, Robert J Hilsden and Darren R Brenner in Journal of Medical Screening
