# Supplementary material for: The impact of episodic screening interruption: COVID-19 and population-based cancer screening in Canada
Source: J Med Screen. 2020 Nov 26;28(2):100–7. doi: 10.1177/0969141320974711 (PMC7691762; doi:10.1177/0969141320974711)
Supplement: sj-pdf-1-msc-10.1177_0969141320974711 - Supplemental material for The impact of episodic screening interruption: COVID-19 and population-based cancer screening in Canada [file sj-pdf-1-msc-10.1177_0969141320974711.pdf]

The impact of episodic screening interruption: COVID-19 and population-based cancer screening in Canada

Supplementary Materials

Contents

Breast cancer Model 4
Occult tumour onset 5
Tumour growth 6
Tumour spread 6
Metastasis 7
Clinical detection 7
Stage at detection 7
Disease progression 7
Breast Cancer Survival 8
Colorectal cancer Model 11
Natural history of colorectal cancer 11
Colorectal cancer screening follow-up of abnormal tests 13
Colorectal cancer survival 14
Screening Regimens 17
References 18

Tables

Supplementary Table 1. Specificity of mammography screening by screen sequence and age..... 4
Supplementary Table 2. Distribution of tumour by age 5
Supplementary Table 3. Annual probability of clinical detection by tumour size 7
Supplementary Table 4. Net breast cancer survival (OncoSim vs. Canadian Cancer Registry) 8
Supplementary Table 5. Distribution of polyp and site by sex, age, and whether the polyp is villous.. 11
Supplementary Table 6. Sensitivity inputs for distal (descending, sigmoid, rectum) and proximal colon (cecum, ascending, transverse) according to screening tool[5]... 12
Supplementary Table 7. Complications of colonoscopy - event probabilities per colonoscopy .. 13
Supplementary Table 8. Net colorectal cancer survival (OncoSim vs. Canadian Cancer Registry) 14
Supplementary Table 9. Colorectal model inputs 15
Supplementary Table 10. Breast and colorectal cancer screening regimens simulated by risk group from the OncoSim microsimulation models... 17

Figures

Supplementary Figure 1. Schematic diagram of the OncoSim-Breast model 4
Supplementary Figure 2. Incidence of 2mm occult tumours by age and time period 5
Supplementary Figure 3. Tumour growth curves (mean growth rate and mean max size by tumour type, class and age)... 6
Supplementary Figure 4. Projected average survival time (years) by age and stage at diagnosis.. 9
Supplementary Figure 5. OncoSim's projected overall survival after breast cancer diagnosis.... 10
Supplementary Figure 6. Schematic diagram of the OncoSim-Colorectal model[4] 12
Supplementary Figure 7. OncoSim's projected overall survival after colorectal cancer diagnosis (A) colon cancer; (B) rectal cancer 14
Supplementary Figure 8. Number of first screens and rescreens for colorectal cancer with and without screening interruption and catch-up scenarios.. 16

Breast cancer Model

Supplementary Table 1. Specificity of mammography screening by screen sequence and age.

Screen sequence	Time since previous screen	Age group				
		Under 40	40-49	50-59	60-69	70+
First screen	-	0.856	0.856	0.876	0.894	0.916
Subsequent screen	< 30 months	0.937	0.937	0.945	0.952	0.956
	> 30 months	0.912	0.912	0.919	0.926	0.930

Source: *Calibration started with estimates from Coldman et al. to match the 2008 abnormal call rate reported in the Canadian Breast Cancer Screening Database.*[1]

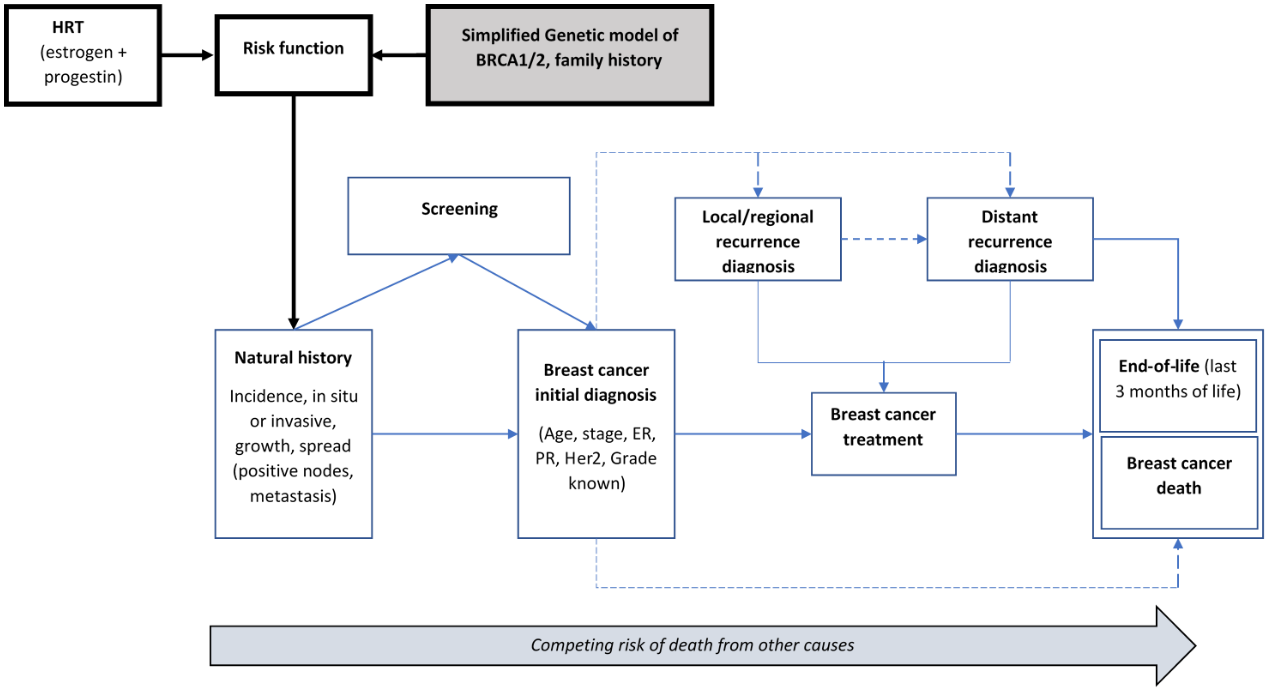

Supplementary Figure 1. Schematic diagram of the OncoSim-Breast model

Occult tumour onset

We simulated the risk of developing an occult tumour for different age groups (Supplementary Figure 2) by calibrating the data from the University of Wisconsin Breast model[2] to match the incidence data in the National Cancer Incidence Reporting System (1969-1991) and the Canadian Cancer Registry (1992-2013). After a tumour is simulated, the model assigns the tumour type (DCIS vs. invasive) by age (Supplementary Table 2).

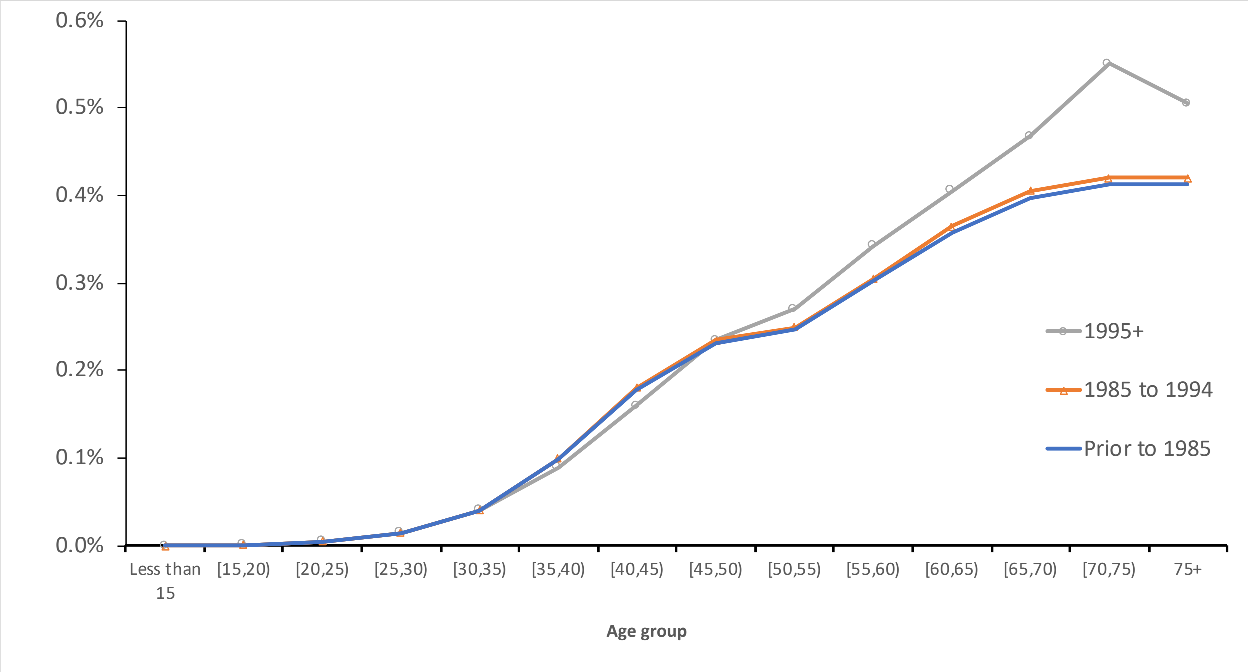

Supplementary Figure 2. Incidence of 2mm occult tumours by age and time period

Supplementary Table 2. Distribution of tumour by age

**Age**	**20-54**	**55-64**	**65-69**	**70-79**	**80+**
**DCIS**	19%	10%	16%	11%	2%
**invasive**	81%	90%	84%	89%	98%

Source: Calibration

Tumour growth

Tumours grow according to years since tumour onset, the presence of BRCA1/2 gene mutation, tumour type (DCIS or invasive) and tumour growth aggressiveness (non-aggressive or aggressive) (Supplementary Figure 3). The growth curves were calibrated from the Wisconsin Breast model's parameters[2] to match stage-specific incidence data in Canadian Cancer Registry (1992-2013) and Canadian Breast Cancer Screening Database (2007-2008) and various other targets.

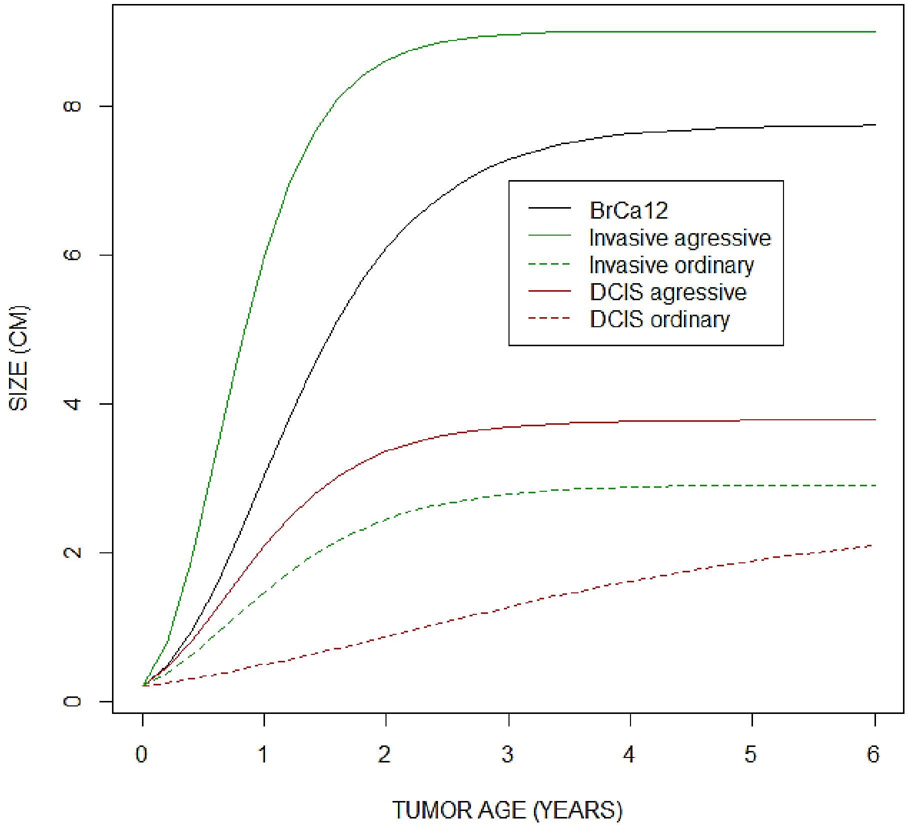

Supplementary Figure 3. Tumour growth curves (mean growth rate and mean max size by tumour type, class and age)

Tumour spread

The spread of an invasive tumour into lymph nodes is determined by size and growth rate of the tumour, and years since tumour onset. The tumour spread equation was developed using data from the University of Wisconsin Breast model[2] and calibrated to match data in the Canadian Cancer Registry (1992-2013) and Canadian Breast Cancer Screening Database (2007-2008).

Metastasis

Risk of metastasis, cancer spreading to places beyond the breast, depends on the tumour size and number of positive nodes. The risk was calibrated to match stage-specific incidence data in Canadian Cancer Registry (1992-2013) and Canadian Breast Cancer Screening Database (2007-2008).

Clinical detection

The probability of clinical detection varies by tumour size (Supplemental Table 3). It was calibrated from the inputs in the Wisconsin Breast model to match the incidence data in the National Cancer Incidence Reporting System (1969-1991) and the Canadian Cancer Registry (1992-2013).

Supplementary Table 3. Annual probability of clinical detection by tumour size

**Tumour size (cm)**	**0.2**	**0.3**	**0.9**	**1.4**	**1.9**	**2.8**	**3.7**	**4.7**	**7.5**	**8.4**
**Probability of tumour getting detected clinically, per year**	0.7%	0.7%	7%	8%	30%	55%	75%	80%	100%	100%

Stage at detection

Once tumour has been detected, stage is assigned based on tumour size (T), nodal status (N), and metastasis (M), according to the American Joint Committee on Cancer (AJCC)'s version 7 classification.

T: The model assigns T (in TNM) at the time of detection. First, it takes a random draw to determine whether it is a T4 tumour. Probability of a T4 tumour (have extended to the chest and/or skin) is a function of age, tumour size T*, number of nodes N* and metastatic status M. Next, it estimates T based on T* (e.g. 2cm < T* < 5cm T=T2) for non-T4 tumours.

N: The model assigns nodal status (N in TNM) at the time of detection from a distribution that depends on age, N*, and T, fitted using the Canadian Cancer Registry data.

Disease progression

Upon cancer detection, the model draws time to disease progression (recurrence and breast cancer death), based on stage, tumour biology, age at diagnosis, and detection method (clinically or screening). A

woman will die from breast cancer if the simulated time to breast cancer death is sooner than the simulated time to non-breast cancer death. We modeled disease progression using data from a cohort of women diagnosed with breast cancer in British Columbia between 2006 and 2009 and followed up until 2014. We fitted the stage-specific outcomes data (diagnosis to local recurrence, diagnosis to distant recurrence, local recurrence to distant recurrence, etc.) to Weibull regression models. Supplemental Figure 4 shows the average simulated survival time by stage and age; Supplemental Figure 5 shows the simulated average overall survival by stage. To adjust for the difference in outcomes between provinces, we applied a relative risk estimate to each province (with British Columbia being the reference, i.e. RR=1); the relative risk estimates were calibrated to fit the survival data in the Canadian Cancer Registry; the relative risk estimates were calibrated to match the survival data in the Canadian Cancer Registry.

Breast Cancer Survival

As an external validation exercise, we compared OncoSim's projected net cancer survival with the latest data from the Canadian Cancer Statistics report. OncoSim's projected net breast cancer survival was considered close to the Canadian Cancer Registry data, when interpreted with the following considerations:

- The latest available net survival reported in the 2019 Canadian Cancer Statistics report[3] referred to data in 2012-2014, while OncoSim's projections shown in the Supplemental Table 4 were for 2020-2029. Breast cancer survival had improved over time due to advancements in treatments and screening.
- OncoSim's projections included *in-situ* carcinomas, but the reporting of *in-situ* carcinomas was incomplete in the Canadian Cancer Registry for cases diagnosed in the earlier years.

Supplementary Table 4. Net breast cancer survival (OncoSim vs. Canadian Cancer Registry)

	**OncoSim**	**CCS 2019***
		**Mean (95% confidence interval)**
**5-year net survival**	93%	88% (88-89)
**10-year net survival**	88%	82% (81-83)

*Data from 2012-2014, excluding Quebec, does not include in situ cases for Ontario diagnosed prior to 2010

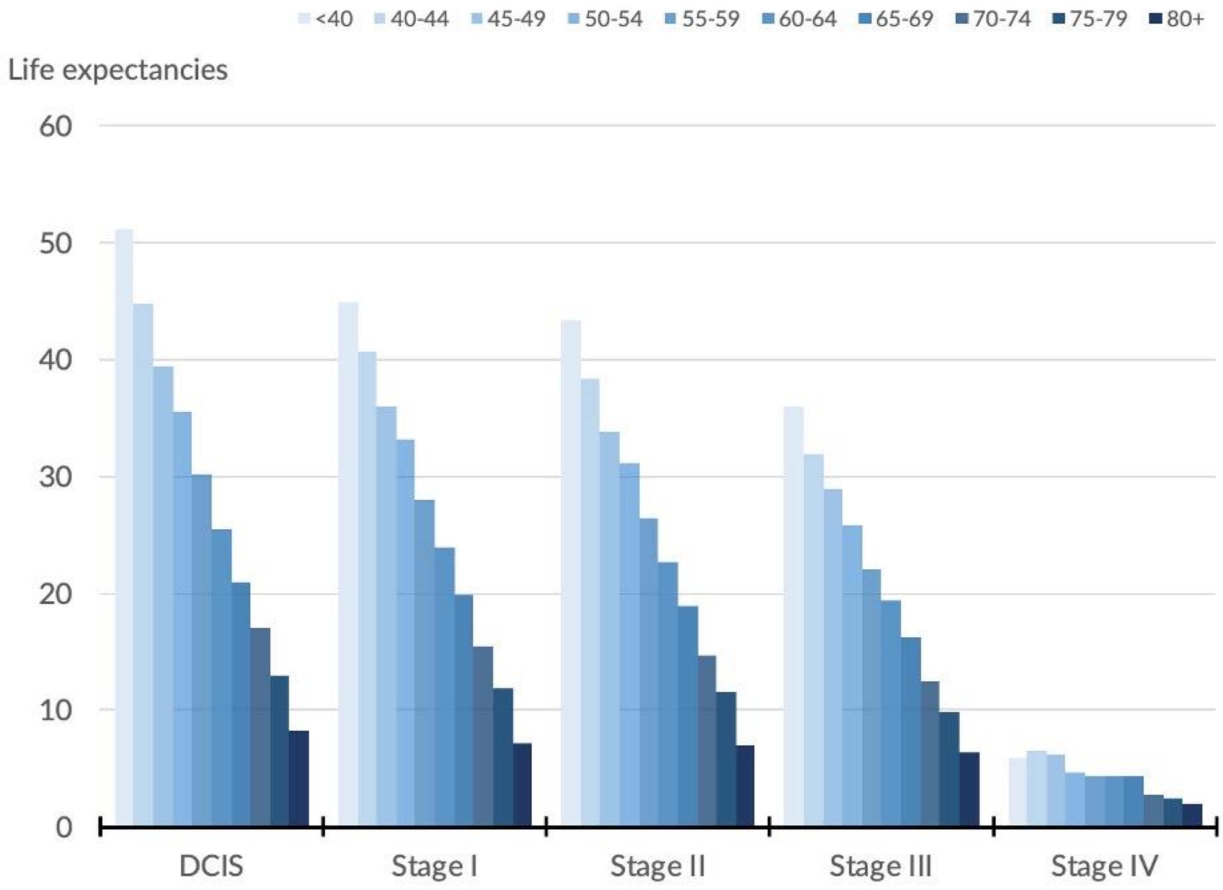

Supplementary Figure 4. Projected average survival time (years) by age and stage at diagnosis

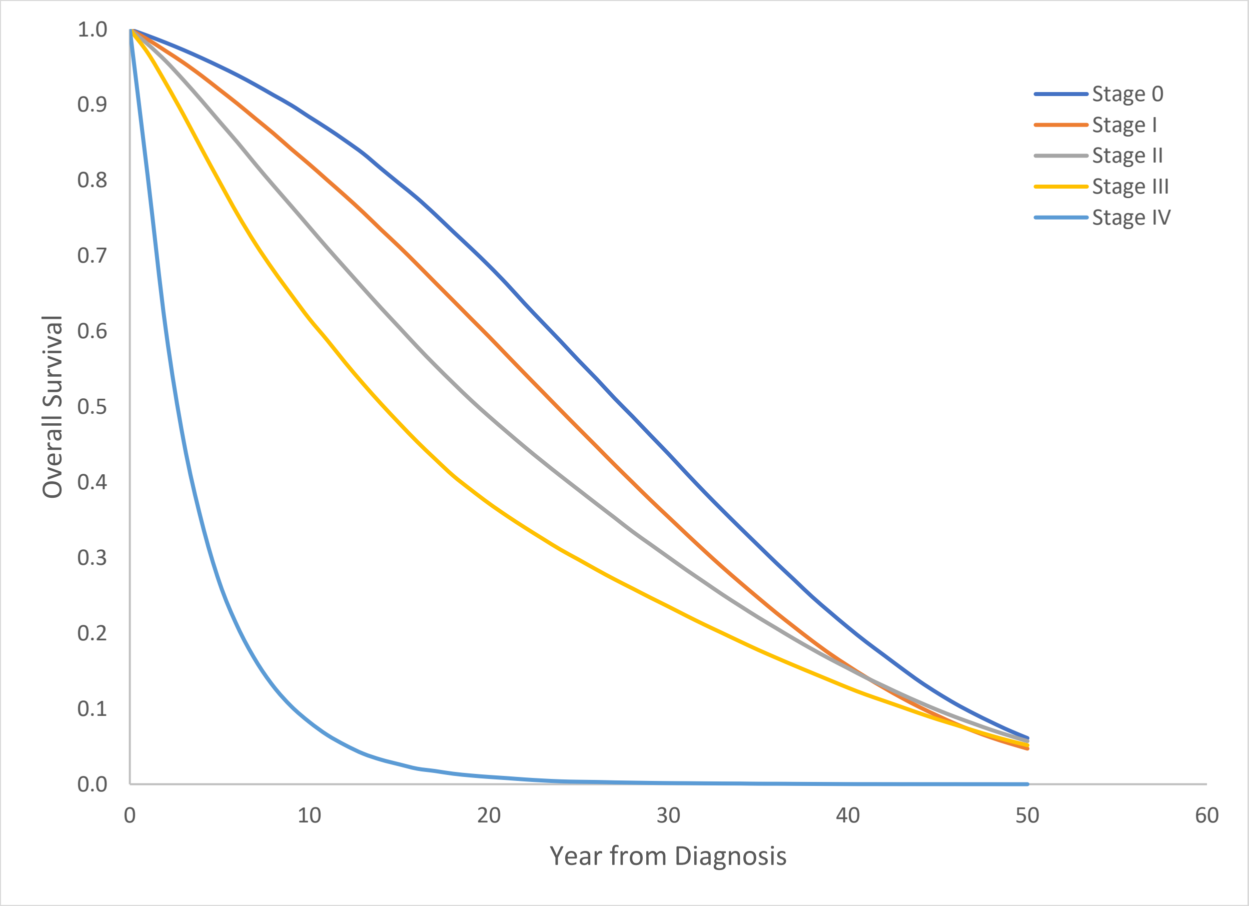

Supplementary Figure 5. OncoSim's projected overall survival after breast cancer diagnosis

Data source: Calibrated to match data in the Canadian Cancer Registry

Colorectal cancer Model

Natural history of colorectal cancer

The model simulates the development of polyps in various sites (cecum, ascending, transverse, descending, sigmoid and rectum) using a distribution (Supplementary Table 5); the probability varies by age and sex. The estimates came from a literature review on adenomatous polyp prevalence, incidence, growth rates, variation by sex, size, site distribution, and histology.[5]

Supplementary Table 5. Distribution of polyp and site by sex, age, and whether the polyp is villous

Sex	Age	Cecum	Ascending	Transverse	Descending	Sigmoid	Rectum
				Non-villous adenoma			
**Female**	[min,60[	0.03	0.32	0.22	0.13	0.19	0.10
	[60,70[	0.05	0.25	0.30	0.07	0.25	0.07
	[70,80[	0.06	0.25	0.32	0.12	0.19	0.06
	[80,max]	0.09	0.25	0.34	0.12	0.18	0.02
**Male**	[min,60[	0.02	0.23	0.16	0.18	0.27	0.14
	[60,70[	0.04	0.19	0.23	0.10	0.34	0.10
	[70,80[	0.05	0.21	0.27	0.15	0.24	0.08
	[80,max]	0.09	0.25	0.34	0.12	0.18	0.02
				Villous adenoma*			
**Female**	[min,60[	0.02	0.19	0.13	0.20	0.31	0.16
	[60,70[	0.03	0.14	0.17	0.12	0.42	0.12
	[70,80[	0.03	0.13	0.17	0.21	0.34	0.11
	[80,max]	0.04	0.12	0.17	0.25	0.38	0.04
**Male**	[min,60[	0.02	0.19	0.13	0.20	0.31	0.16
	[60,70[	0.03	0.14	0.17	0.12	0.42	0.12
	[70,80[	0.03	0.13	0.17	0.21	0.34	0.11

	[80,max]	0.04	0.12	0.17	0.25	0.38	0.04

* This category includes tubulovillous adenomas because those adenomas are tubular and villous.

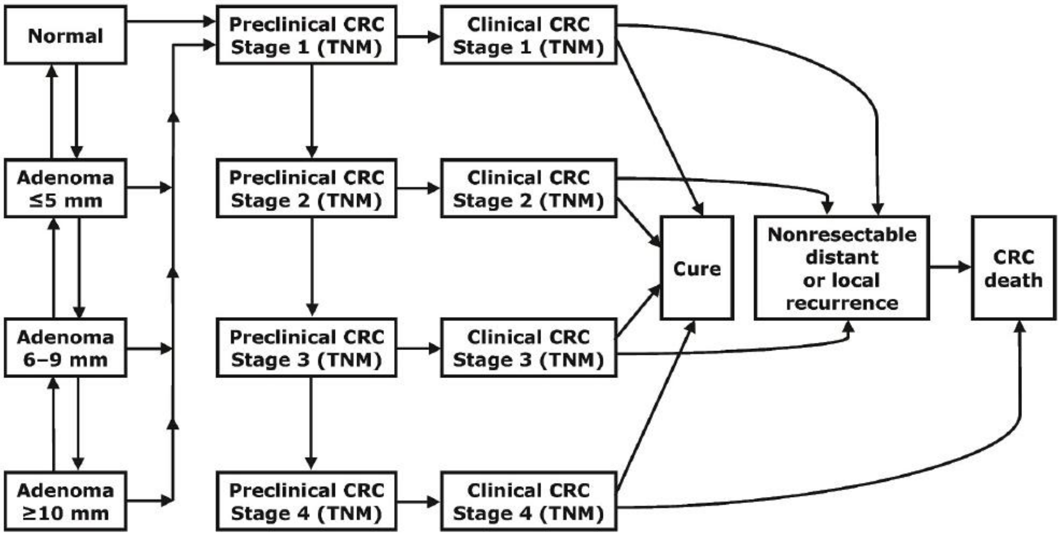

Supplementary Figure 6. Schematic diagram of the OncoSim-Colorectal model[4]

Supplementary Table 6. Sensitivity inputs for distal (descending, sigmoid, rectum) and proximal colon (cecum, ascending, transverse) according to screening tool[5]

	**FIT immunochemical**	**Colonoscopy**
Polyp less or equal to 5mm in size	0.04	0.75
Polyp between 6 and 9mm in size	0.1	0.85
Polyp greater or equal to 10mm in size	0.3	0.95
Cancer	0.75	0.95

Supplementary Table 7. Complications of colonoscopy - event probabilities per colonoscopy[6,7,8]

**Complications of colonoscopy**	**Event rate per colonoscopy**
Death	0.0002
Perforation	0.0017
Haemorrhage	0.0003
Infection	0
Cardiopulmonary event	0

Colorectal cancer screening follow-up of abnormal tests

After colonoscopy investigation, subjects are classified into four groups: adenoma-free, low risk, high risk, and cancer. Low risk subjects have fewer than 3 small (<10 mm) nonvillous adenomas and receive another colonoscopy in 5 years; if clear, they then return to screening. High-risk subjects, defined as having 3 or more small adenomas, 1 or more large adenomas (≥10 mm) or an adenoma with a villous or tubulovillous component, receive colonoscopies 3 years after the first follow-up colonoscopy and 5 years after the subsequent colonoscopy. Subjects with cancer receive a colonoscopy the next year and every 3 years thereafter. All adenomas identified at colonoscopy are assumed to be successfully treated.

Colorectal cancer survival

OncoSim's projected overall survival by stage for colon and rectal cancers are shown in Supplementary Figure 7. As an external validation exercise, we compared OncoSim's projected net cancer survival with the latest Canadian Cancer Registry data in the Canadian Cancer Statistics report[3]. OncoSim's projections were similar to the observed estimates (Supplementary Table 8).

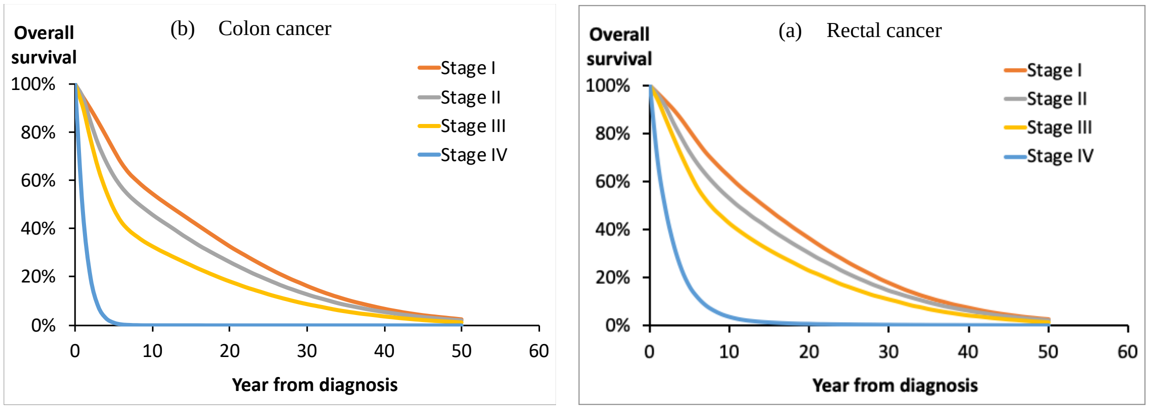

Supplementary Figure 7. OncoSim's projected overall survival after colon cancer diagnosis (a) colon cancer; (b) rectal cancer

Data source: Calibrated to match data in the Canadian Cancer Registry

Supplementary Table 8. Net colorectal cancer survival (OncoSim vs. Canadian Cancer Registry)

	**OncoSim**	**CCS 2019***
		**Mean (95% confidence interval)**
**5-year net survival**	66%	65% (65-66)
**10-year net survival**	59%	60% (59-61)

**Data from 2012-2014, excluding Quebec*

Supplementary Table 9. Colorectal model inputs

**Variable**	**Estimate**	**Source**
Colorectal cancer FIT screening participation rate*	42.3%	CCHS [9]
Adherence rate for follow-up colonoscopy after FIT	85%	CCHS [9]
Colonoscopy screening participation rate for individuals with family history of colorectal cancer**	40%	Assumption
**Sensitivity of FIT (threshold: 100ng/mL)**		
Small adenoma (<=5 mm)	0.04	Coldman et al. (2015) [5]
Medium adenoma (6-9 mm)	0.1	Coldman et al. (2015) [5]
Large adenoma (>=10 mm)	0.3	Coldman et al. (2015) [5]
Cancer	0.75	Coldman et al. (2015) [5]
Specificity of FIT	0.96	Coldman et al. (2015) [5]
**Sensitivity of colonoscopy**		
Small adenoma (<=5 mm)	0.75	Coldman et al. (2015) [5]
Medium adenoma (6-9 mm)	0.85	Coldman et al. (2015) [5]
Large adenoma (>=10 mm)	0.95	Coldman et al. (2015) [5]
Cancer	0.95	Coldman et al. (2015) [5]
Specificity of colonoscopy	1	Coldman et al. (2015) [5]

*First time recruitment rate of 53% and 80% rescreen rate for an overall participation rate of ~42.3%.
** Assumed 50% of those with family history of colorectal cancer would receive colonoscopy screening, and 80% of them would participate in subsequent screening (every 5 years). The remaining persons with family history would be recruited to participate in the average risk FIT screening program (see participation rate above).

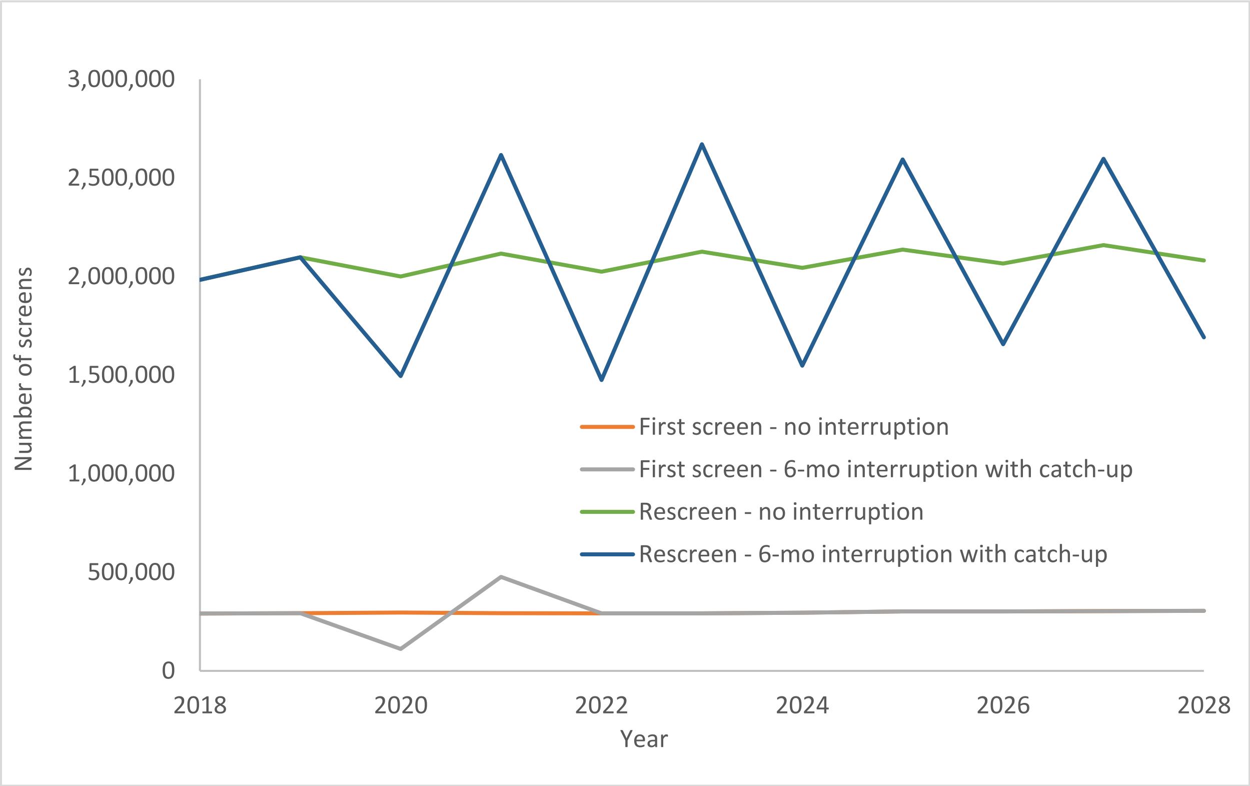

Supplementary Figure 8. Number of first screens and rescreens for colorectal cancer with and without screening interruption and catch-up scenarios

Screening Regimens

Supplementary Table 10. Breast and colorectal cancer screening regimens simulated by risk group from the OncoSim microsimulation models

	**Breast**		**Colorectal**	
**Risk group**	**Average**	**Elevated***	**Average risk**	**Elevated****
**Test**	Digital mammogram	Digital mammogram	Fecal immunochemical test (FIT)	Colonoscopy
**Interval**	2 years	1 year	2 years	5 years
**Screening ages**	50-74 years	40-74 years	50-74 years	40-74 years

Abbreviations: FIIT, fecal immunochemical test
* Women with family history of breast cancer and/or BRCA 1/2 mutation; we assumed 65% of these women are screened with this regimen, and the remaining 35% are invited to screen with the average risk regimen.
** People with one or more first degree family history of colorectal cancer; we assumed 50% of elevated risk individuals are screened with this regimen, and the remaining 50% are invited to screen with the average risk regimen.

References

1. Coldman AJ, Phillips N. False-positive Screening Mammograms and Biopsies Among Women Participating in a Canadian Provincial Breast Screening Program. Canadian Journal of Public Health 2012; 103(6): e420-e4.
2. Alagoz O, Ergun MA, Cevik M, et al. The University of Wisconsin Breast Cancer Epidemiology Simulation Model: An Update. Med Decis Making 2018; 38(1_suppl): 99s-111s.
3. Canadian Cancer Statistics Advisory Committee. Canadian Cancer Statistics 2019. Toronto, ON: Canadian Cancer Society; 2019. Available at: cancer.ca/Canadian-Cancer-Statistics-2019-EN (accessed July 1, 2020).
4. Coldman A, Flanagan W, Nadeau C, Wolfson M, Fitzgerald N, Memon S, et al. Projected effect of fecal immunochemical test threshold for colorectal cancer screening on outcomes and costs for Canada using the OncoSim microsimulation model. J Cancer Policy. 2017 Sep;13:38–46.
5. Coldman AJ, Phillips N, Brisson J, et al. Evaluating colorectal cancer screening options for Canada using the Cancer Risk Management Model. Curr Oncol. 2015 Apr; 22(2):e41-50.
6. Habr-Gama A, Waye JD. Complications and hazards of gastrointestinal endoscopy. World J Surg. 1989;13(2):193-201. doi:10.1007/BF01658399
7. Bercy G, et al.: Complications of colonoscopy and polypectomy. Gastroent. 1974. 67: 548.
8. Macrae FA, Tan KG, Williams CB: Towards safer colonoscopy: a report on the complications of 5000 diagnostic or therapeutic colonoscopies. Gut 1983; 24:376-383.
9. Canadian Community Health Survey (CCHS) 2018, Statistics Canada, available from: https://www23.statcan.gc.ca/imdb/p3Instr.pl?Function=assembleInstr&a=1&&lang=en&Item_Id=839130
